# Results of the “GER-e-TEC” Experiment Involving the Use of an Automated Platform to Detect the Exacerbation of Geriatric Syndromes

**DOI:** 10.3390/jcm9123836

**Published:** 2020-11-26

**Authors:** Abrar-Ahmad Zulfiqar, Orianne Vaudelle, Mohamed Hajjam, Bernard Geny, Samy Talha, Dominique Letourneau, Jawad Hajjam, Sylvie Erve, Amir Hajjam El Hassani, Emmanuel Andrès

**Affiliations:** 1 Service de Médecine Interne, Diabète et Maladies Métaboliques de la Clinique Médicale B, Hôpitaux Universitaires de Strasbourg et Equipe EA 3072 “Mitochondrie, Stress Oxydant et Protection Musculaire”, Faculté de Médecine-Université de Strasbourg, 67000 Strasbourg, France; emmanuel.andres@chru-strasbourg.fr; 2 Predimed Technology Society, 67300 Schiltigheim, France; orianne.vaudelle@predimed-technology.com (O.V.); mohamed.hajjam@predimed-technology.com (M.H.); 3 Service de Physiologie et d’Explorations Fonctionnelles, Hôpitaux Universitaires de Strasbourg et Equipe EA 3072 “Mitochondrie, Stress Oxydant et Protection Musculaire”, Faculté de Médecine-Université de Strasbourg, 67000 Strasbourg, France; bernard.geny@chru-strasbourg.fr (B.G.); samy.talha@chru-strasbourg.fr (S.T.); 4 Fondation de l′Avenir pour la Recherche Médicale Appliquée Research Department, 75015 Paris, France; dletourneau@fondationdeavenir.org; 5 Centre d′Expertise des TIC pour l′autonomie (CenTich) et Mutualité Française Anjou-Mayenne (MFAM)-Angers, 49000 Angers, France; jawad.hajjam@centich.fr (J.H.); sylvie.erve@centich.fr (S.E.); 6 Laboratoire IRTES-SeT, Université de Technologie de Belfort-Montbéliard (UTBM), Belfort-Montbéliard, 90000 Belfort, France; amir.hajjam@utbm.fr

**Keywords:** remote monitoring, geriatric risks, MyPredi™ platform, GER-e-TEC study, prevention, elderly patients

## Abstract

Introduction: Telemedicine is believed to be helpful in managing patients suffering from chronic diseases, in particular elderly patients with numerous accompanying conditions. This was the basis for the “GERIATRICS and e-Technology (GER-e-TEC) study”, which was an experiment involving the use of the smart MyPredi™ e-platform to automatically detect the exacerbation of geriatric syndromes. Methods: The MyPredi™ platform is connected to a medical analysis system that receives physiological data from medical sensors in real time and analyzes this data to generate (when necessary) alerts. These alerts are issued in the event that the health of a patient deteriorates due to an exacerbation of their chronic diseases. An experiment was conducted between 24 September 2019 and 24 November 2019 to test this alert system. During this time, the platform was used on patients being monitored in an internal medicine unit at the University Hospital of Strasbourg. The alerts were compiled and analyzed in terms of sensitivity, specificity, and positive and negative predictive values with respect to clinical data. The results of the experiment are provided below. Results: A total of 36 patients were monitored remotely, 21 of whom were male. The mean age of the patients was 81.4 years. The patients used the telemedicine solution for an average of 22.1 days. The telemedicine solution took a total of 147,703 measurements while monitoring the geriatric risks of the entire patient group. An average of 226 measurements were taken per patient per day. The telemedicine solution generated a total of 1611 alerts while assessing the geriatric risks of the entire patient group. For each geriatric risk, an average of 45 alerts were emitted per patient, with 16 of these alerts classified as “low”, 12 classified as “medium”, and 20 classified as “critical”. In terms of sensitivity, the results were 100% for all geriatric risks and extremely satisfactory in terms of positive and negative predictive values. In terms of survival analysis, the number of alerts had an impact on the duration of hospitalization due to decompensated heart failure, a deterioration in the general condition, and other reasons. Conclusion: The MyPredi™ telemedicine system allows the generation of automatic, non-intrusive alerts when the health of a patient deteriorates due to risks associated with geriatric syndromes.

## 1. Introduction

According to the INSEE (Institut National de la Statistique et des Etudes Economiques, Paris, France) [1], 25.6% of people in France are aged 60 and over. Furthermore, 600,000 people with a mean age of 85 live in nursing homes. Nursing home patients are usually polypathological (heart failure, hypertension, malnutrition, diabetes, chronic obstructive pulmonary disease (COPD), kidney failure, cognitive and psychobehavioral disorders) and are polymedicated. Older people enter nursing homes later in their lives, and when they do, they are most often dependent, as shown in a study conducted by Morley in 2011. In that study, the proportion of residents classified from 4 to 1 in the Iso-Resource Group (GIR) represented 91% of cases [2].

From a medical standpoint, these data demonstrate the need for regular patient monitoring and a high level of medical (and even multidisciplinary) expertise on the part of healthcare teams. Furthermore, the risks associated with the geriatric syndromes of elderly patients must also be taken into consideration [3]. These risks include pain, falls, constipation, dehydration, confusion, iatrogenesis, malnutrition, heart failure (HF), hypertension, diabetes, infections, bedsores, psychobehavioral disorders, etc. [3]. For the elderly, these conditions usually result in the need for hospitalization or institutionalization. As far as we know, no telemedicine project to date has allowed for the monitoring and detection of all these risks.

In this context, we tested the MyPredi™ e-platform in an internal medicine unit. This platform is dedicated to the automated and intelligent detection of situations at risk of geriatric syndromes.

This article describes the protocol of a pilot study to evaluate the feasibility of the MyPredi™ remote monitoring program, allowing for the long-term, comprehensive, evidence-based monitoring of multimorbid seniors. The main goal of the GER-e-TEC study is to evaluate the use of a remote monitoring platform as a means of structuring and standardizing the medical care of dependent elderly patients to prevent acute exacerbations and other complications associated with geriatric syndromes. Below, we present the results of a pilot study involving 36 elderly patients who were hospitalized with an acute disease. To our knowledge, this is the very first study to use a remote monitoring platform to detect and monitor geriatric syndromes.

## 2. Patients and Method

### 2.1. Objective

During the GER-e-TEC project, a pilot study was conducted in a hospital setting to evaluate the use of the MyPredi™ remote monitoring platform, to not only monitor the chronic diseases of elderly patients (hypertension, heart failure, diabetes, kidney failure, chronic obstructive pulmonary disease (COPD), etc.) but also to detect the risks and disorders associated with these syndromes. The platform had already proven effective at monitoring heart failure (HF) and diabetes [4].

The goal of our work is to develop a codified preventive approach for the management of the main geriatric risks using a personalized remote monitoring platform in order to avoid factors leading to acute decompensation in the elderly.

### 2.2. Patients

The study was conducted in the Department of Internal Medicine, Diabetes, and Metabolic Disorders at the University Hospital of Strasbourg (HUS, Strasbourg, France) from 24 September 2019 to 24 November 2019. Any patient over 65 years of age admitted to the hospital or emergency room with one or more chronic diseases was eligible for the GER-e-TEC study. Minors, pregnant women, patients who were unable to sign the eligibility and consent form, elderly patients in palliative care (i.e., patients in hospice with life-limiting diseases), and patients who refused to provide their consent were excluded from the study. If patients presented signs of severe dementia, the consent of their legal guardian was required.

### 2.3. Study Outline

During the experiment, patients and healthcare professionals used the MyPredi™ remote monitoring platform daily to collect physiological data via connected sensors. Therefore, the patients benefited from a remote monitoring solution in addition to their usual care. The collected data were automatically sent to the platform for analysis. Moreover, questionnaires were regularly completed by the medical and paramedical staff. These questionnaires were analyzed and used to complement the monitoring of the general health, chronic diseases, and geriatric syndromes of the patient.

In addition to collecting and making data available to the caregivers, the remote monitoring platform made it possible to quickly detect anomalies in the patient′s health and send alerts to healthcare professionals. However, the alerts were not sent to the patients or their direct caregivers in order to comply with the study protocol (“routine care”) and the recommendations of the ethics committee.

During the experiment, the alerts were compiled in the order in which they were received. They were analyzed with regard to the clinical context at the time they were emitted, using the discharge letter and computer files (medical and nursing) of the patient in question. This analysis was performed retrospectively by two professionals involved in the present study but not in contact with the caregivers who cared for the patient on a daily basis. The alerts were classified as “relevant” or “irrelevant”, i.e., whether or not they were associated with an action or intervention by the clinic.

All alerts were processed and were then analyzed and labeled “relevant” in light of an avoided clinical event, a decided hygienic–dietetic or therapeutic modification made. Thus, each “alert” was classified as “relevant” or not, in relation (or not) to an event or clinical fact interfering that may result in or signaling.

The “onset or aggravation of cardiac decompensation” is that which requires medical intervention (therapeutic adaptation) or which will quickly (in a few days) lead to hospitalization.

A “deterioration of the patient′s state of health and other reason” is defined by a decompensation or an aggravation of chronic pathologies (chronic HF, diabetes mellitus, COPD, etc.) that requires medical intervention (therapeutic adaptation) or will quickly (in a few days) lead to hospitalization.

The severity level of the so-called “low, moderate, and critical” alerts was defined according to the opinion of the medical expert committee piloting the “GER-e-TEC” study, bringing together geriatricians, internists, and cardiologists. The alerts were analyzed retrospectively by two experts with the help of the patient′s medical and paramedical file.

## 3. Experimental Protocol

**Figure 1:**
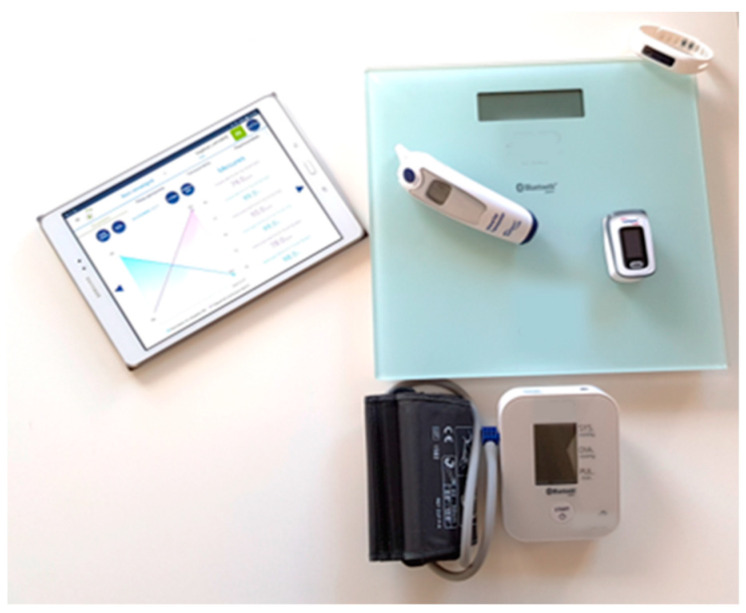
Devices used within the framework of the MyPredi™ platform [3].

The MyPredi™ solution (i.e., a tablet and connected sensors) (Figure 1) was used to collect the patient’s physiological data, including blood pressure, heart rate, weight, oxygen saturation, capillary blood glucose, and temperature three times per day (morning, noon, and night). A number of physiological measurements were taken by the pedometer for physical activity and sleep. The patient wore the pedometer day and night, and the data (physical activity and sleep) were automatically sent to the MyPredi™ platform.

Additional information on geriatric risks and disorders was collected on a daily basis by way of questionnaires completed on the tablet. These questionnaires addressed falls, constipation, dehydration, confusion, iatrogenesis, malnutrition, heart failure, hypertension, diabetes, infections, and bedsores. A therapy-related questionnaire was also completed by the caregivers in conjunction with the patient during the patient′s stay. Table 1 illustrates the geriatric risks and disorders that were monitored during the experiment. Table 2 and Table 3 illustrate the detailed questionnaires embedded within the MyPredi™ platform and used for monitoring the geriatric risks studied.

## 4. The Remote Monitoring Platform

The MyPredi™ remote monitoring platform is a “smart” connected platform. It has been tested as a remote monitoring tool for patients with heart failure, hypertension, and diabetes [4]. MyPredi™ is ISO 13485 certified and has been awarded a CE mark [3]. The current version of the MyPredi™ (Figure 1) used in the context of the GER-e-TEC project is comprised of the following.

- Non-intrusive medical sensors (blood pressure, heart rate, oxygen saturation, weight, physical activity) that communicate via Bluetooth, allowing for the collection of real-time physiological data on the general health and chronic disease (hypertension, heart rate, diabetes, etc.) of patients. See Table 4 for more detailed information on the sensors.
- A tablet that communicates via Wi-Fi or 3G/4G, allowing healthcare professionals to interact with patients and provide information on hygiene, nutrition, and physical therapy (not available at the time of the experiment).
- Medical questionnaires addressing the primary geriatric risks and disorders such as pain (as assessed by a visual analogue scale (VAS), VRS (verbal rating scale), and Algoplus scale), constipation (frequency of bowel movements), dehydration (amount of daily water intake), iatrogenic risk, heart failure, as well as sleep quality and bed rest (bedsores/physical activity). These questionnaires were also used to collect certain biological data, e.g., natremia, kalemia, albuminemia, International Normalized Ratio (INR), and digoxinemia. These data are useful when monitoring certain conditions such as kidney failure and assessing the patient’s hydration level.
- A hosting Internet server (French health authorities accredited medical provider): patient data, an “intelligent” system in the form of an inference engine and a medical ontology, allowing the personalized analysis of data specific to each patient in real time or delayed, ultimately with the generation of “alerts”.
- A secure Internet portal (website), allowing the patient and various health professionals (referring doctor, cardiologist, nurse, etc.) to log in according to their right of access.

The MyPredi™ platform uses an ontology that defines a controlled vocabulary (pathologies, drugs, symptoms, etc.) and models the concepts relating to the monitoring of chronic pathologies. The effective use of an ontology for reasoning purposes requires the addition of operational semantics that specify how the knowledge modeled in the ontology will be used to reason and automatically produce new knowledge. The reasoning part is based on rules that are either introduced by the medical experts (here, cardiologists, internists, geriatricians) or generated by data mining and validated by medical experts. These are not exposed here because they are covered by the patent. The medical knowledge is derived from “evidence-based medicine”.

The MyPredi™ platform sends an alert to the caregivers if any of the data from the sensors or questionnaires deviates from this knowledge. Once an alert is received, the healthcare team can act accordingly. They can verify the relevance of the alert, make sure the measurement protocol was respected, verify the patient’s hygiene, diet, and medication, and adjust the treatment or accompanying measures (an increase in activity, prevention of bedsores, attention to diet, etc.). As mentioned above, this part was not included in the pilot study, as caregivers did not receive the alerts in order to comply with the experimental protocol. The MyPredi™ platform generates indicators of deterioration of the patient′s health, which are called “alerts”, in connection with a decompensation of chronic pathologies. These are related to an interfering, proven, and documented fact or medical event, or even an intercurrent medical pathology, such as fever, disturbance of blood pressure or heart rate, weight gain, lung infection, non-adherence to therapy, etc. In principle, these indicators exclude “vital alerts” such as chest pain, the paralysis of a limb, etc., which are not affected by the MyPredi™ platform. In this context, the patient and the healthcare professionals in charge of the patient are informed that the usual emergency procedures must be followed.

The alert level (low, medium, critical) was defined by the medical knowledge derived from “evidence-based medicine” in conjunction with the main academic associations and medical experts involved in the study (cardiologists, internists, geriatricians).

## 5. Parameters Evaluated and Statistical Analyses

To determine the clinical relevance of the alerts generated by the MyPredi™ remote monitoring platform, we calculated the sensitivities (Se), the specificities (Spe), and the positive (PPV) and negative (NPV) predictive values of the alerts with respect to each risk or disorder that was detected.

We also calculated the Charlson Comorbidity Index of the included population.

For the survival analysis, the Kaplan–Meier method was used to estimate the overall survival curve. A logrank test was used to compare the survival curves for the “decompensated heart failure”, “deterioration of general condition”, and “other reasons” groups. “Other reasons” included abdominal pain, erysipelas, confusion, potential biological inflammatory syndrome, deep vein thrombosis, pulmonary infection, asthenia, pneumopathy, kidney failure, urinary retention, melena, hypocalcemia, anemia, pleural effusion, and ischemia.

The test statistic under this hypothesis approaches a chi-square distribution with (number of groups 1) degrees of freedom, with the hypotheses as follows:

**Hypothesis 1** **(H1).** *Equality of survival functions.*

**Hypothesis 2** **(H2).** *Inequality of survival functions.*

We used RStudio (https://rstudio.com/products/rstudio/download/) software and R code (Boston, MA, USA).

## 6. Administrative Requirements

All patients who participated in the GER-e-TEC project were required to sign a consent form. A clinical research protocol for the GER-e-TEC project was filed with the Ethics Committee of the Faculty of Medicine of Strasbourg under RNI 2020-HUS N°7792. We also obtained authorization to conduct the study from the Commission Nationale Informatique et Liberté (CNIL, “National Commission on Informatics and Liberty”, Strasbourg, France).

## 7. Results

### Characteristics of Patients

A total of 81 patients were hospitalized in the internal medicine unit between 24 September 2019 and 24 November 2019. Of these, 36 patients were elderly and agreed to be monitored remotely as part of the pilot study, while 45 patients did not meet the eligibility requirements (43 patients were under 65 years of age and two were in palliative care). The mean age of the patients was 81.4 years with a standard deviation of 7.7 years. The median age was 82.4 years. There were 21 (58.3%) male patients and 15 female patients: a male/female ratio of 1.4 to 1. The patients used the telemedicine solution for an average of 22.1 days with a standard deviation of 18.5. Among the 36 patients, 31 (86.1%) had cardiovascular disease, 23 (64%) had a history of hypertension, 13 (36.1%) had a history of lung disease, 11 (30.5%) had a history of asthma/COPD, 14 (38.8%) had a history of diabetes, and 4 (11.1%) had a history of solid tumors. See Table 5 for more information on the accompanying syndromes. The main reasons for hospitalization were lung infections (17 patients) and decompensated heart failure (10 patients). See Table 5 for more details. The average number of drug treatments at the time of admission was 8.5 with a standard deviation of 4.2. Most of the drugs used for treatment were antihypertensives (*n* = 77). See Table 5 for more information on the treatments. The mean Charlson score was 6.86 (2–14). The average length of stay was 17.5 days (4–43).

Of the 36 patients, 30 lived at home and 6 lived in nursing homes. After hospitalization, 23 patients returned to their homes, 8 went to nursing homes, and 5 were transferred to a rehabilitation center for further treatment. None of the patients died during the experiment.

## 8. Data from the Sensors/Questionnaires

The MyPredi™ remote monitoring solution collected a total of 147,703 measurements while monitoring the geriatric syndromes of the entire patient group. On average, 4476 measurements were recorded per patient for geriatric disorders and risks. On average, 226 measurements were recorded per patient per day.

See Table 6 for details about data from sensors/questionnaires

## 9. Number of Alerts for Geriatric Syndromes and Chronic Diseases

The telemedicine solution emitted a total of 1611 alerts while monitoring the geriatric syndromes of the entire patient group. For each geriatric risk/disorder, an average of 45 alerts were emitted per patient, with 16 of these alerts classified as “low”, 12 classified as “medium”, and 20 classified as “critical”.

During the monitoring (Table 7), 336 alerts (20.9%) were emitted for the “HF” risk, with an average of 11.2 alerts per patient and a standard deviation of 9.3; 290 alerts (18%) were emitted for the “dehydration” risk, with an average of 10 alerts per patient and a standard deviation of 7.9; 274 alerts (17%) were emitted for the “hypertension” risk; 211 alerts (13.1%) were emitted for the “iatrogenic” risk, with an average of 7 alerts per patient and a standard deviation of 10; and 192 alerts (11.9%) were emitted for the “bed rest” risk, with an average of 6.9 alerts per patient and a standard deviation of 4.2. For the other geriatric syndromes components, 137 alerts (8.5%) were emitted for the “pain” risk, with an average of 6.5 alerts per patient and a standard deviation of 8.5; 122 alerts (7.6%) were emitted for the “iatrogenic” risk, with an average of 7 alerts per patient and a standard deviation of 10; 66 alerts (4.1%) were emitted for the “kidney failure” risk, with an average of 5.5 alerts per patient and a standard deviation of 4.1; 66 alerts (4.1%) were emitted for the “hypo or hypernatremia” risk, with an average of 5.5 alerts per patient and a standard deviation of 4.1; and 60 alerts (3.7%) were emitted for the “malnutrition” risk, with an average of 3.2 alerts per patient and a standard deviation of 2.8. Less frequently, 28 alerts (1.7%) were emitted for the “decrease or increase in heart rate” risk, with an average of 2.8 alerts per patient and a standard deviation of 4; 9 alerts (0.6%) were emitted for the “hypo or hyperkalemia” risk, with an average of 3 alerts per patient and a standard deviation of 2.6; 9 alerts (0.6%) were emitted for the “insufficient physical activity” risk, with an average of 3 alerts per patient and a standard deviation of 2.6; and 8 alerts (0.5%) were emitted for the “hyperthermia” risk, with an average of 1.6 alerts per patient and a standard deviation of 0.9. Table 5 illustrates the criticality of the alerts for each of the geriatric syndromes. No alerts were emitted for the “constipation”, “diabetes”, and “bedsore” risks. In the total population, a mean number of 1.8 critical alerts per day per patient was observed.

## 10. Clinical Relevance of Alerts

Table 8 indicates alerts and recommendations issued for the geriatric risks studied. Risks associated with decompensated heart failure (*n* = 336), dehydration (*n* = 290), and iatrogenesis (*n* = 211) generated the most alerts.

Table 9 illustrates the clinical relevance of the alerts in terms of Se, Spe, PPV, and NPV for the evaluated criteria. Note the sensitivity of 100% for the alerts of all the evaluated geriatric risks and the high negative predictive value.

**Figure 2:**
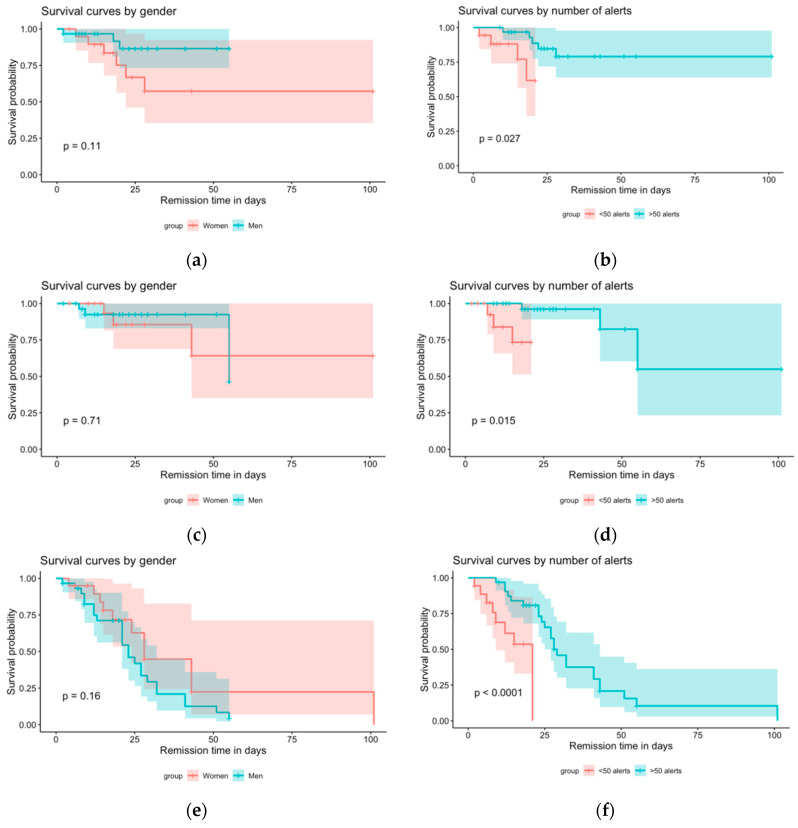
Survival analysis. (**a**): Survival curves by gender following cardiac decompensation. (**b**): Survival curves by number of alerts following cardiac decompensation. (**c**): Survival curves by gender following alteration of general health. (**d**): Survival curves by number of alerts following alteration of general health. (**e**): Survival curves by gender following other reason. (**f**): Survival curves by number of alerts following other reason.

Survival analyses (Figure 2) showed that gender played no role in the length of the hospital stay, regardless of the reason for the hospitalization (decompensated heart failure (*p* = 0.11), deterioration of general condition (*p* = 0.7), other reasons (*p* = 0.2)). However, the analyses revealed that the length of the hospital stay was affected by the number of alerts (decompensated heart failure (*p* = 0.03), deterioration of general condition (*p* = 0.01), and other reasons (*p*-value = 3 × 10^−5^)).

## 11. Discussion

These results show that the MyPredi™ remote monitoring platform is effective at automatically and non-intrusively generating alerts in the event of increased geriatric risks, in particular those associated with pain, heart rate, bed rest, and decompensated heart failure. In fact, the system is most adept at detecting these risks, with sensitivity and positive predictive values of 100%. For the practitioner, this experiment highlights the ability of the MyPredi™ remote monitoring platform to detect and emit alerts for 100% of the above-mentioned risks. The MyPredi™ solution was also shown to be effective at detecting other geriatric risks such as dehydration and malnutrition. As long as no alert is emitted, healthcare teams can rest assured that the patient is doing well (NPV of 100%). In this context, a certain number of false alarms can be expected mainly due to non-compliance with the protocol but also due to the sensitivity of the system. Moreover, the survival analyses of patients hospitalized for “decompensated heart failure”, “deterioration of general condition”, and “other reasons” produced some interesting and innovative results. In terms of the survival analysis, the number of alerts had an impact on the duration of hospitalization due to decompensated heart failure, a deterioration in the general condition, and other reasons. There is a 95% chance that the number of alerts emitted by the tool per patient is indicative of the probability that the patients will not require hospitalization. Consequently, patients receiving more than 50 alerts have a higher chance of surviving than patients receiving fewer than 50 alerts. Therefore, the generation of alerts is beneficial to the patient.

The MyPredi™ remote monitoring system generates automatic and non-intrusive alerts associated with the previously mentioned geriatric risks. This study demonstrates the importance of the technological choices, tools, and solutions developed and adopted in MyPredi™ in the monitoring geriatric patients. All patients and healthcare professionals found the remote monitoring system extremely easy to use throughout the entire experiment. Previously, the MyPredi™ (formerly *E-Care*™) remote monitoring platform was used with elderly patients to detect the risk of “heart failure” with similar results [4]. Therefore, the healthcare team could monitor the patient remotely, providing comprehensive and personalized treatment of the areas of concern detected by the platform and help the patient with their therapy.

Although remote monitoring systems for the elderly are becoming more common, the majority of the work with this age group involves the remote monitoring of heart failure and other cardiovascular problems, mainly in the patient’s home [5,6,7,8,9,10]. Other remote monitoring projects involving the elderly have focused on other chronic diseases, in particular COPD [11,12,13] and diabetes [14].

Guan et al. proposed a remote health monitoring system for the elderly based on a smart home gateway. The proposed system consisted of smart clothing, the smart home gateway, and the health care server. The smart clothing collected the elderly′s electrocardiogram (ECG) and motion signals. The home gateway was used for data transmission. A system demonstration showed that the ECG signals and motion signals of the elderly could be monitored. The proposed system has good scalability and is simple to operate. It has the potential to provide long-term and continuous home health monitoring services for the elderly [15].

A four-year tele-health intervention (the Health Diary system based on digital pen technology) was implemented for advanced COPD for HF in patients over 65 years old. Patients were introduced to the telemonitoring system which was supervised by a specialized hospital-based home care (HBHC) unit. Staff associated with this unit were responsible for the healthcare provided. The study included patients with COPD or HF aged ≥65 years who were frequently hospitalized due to exacerbations, e.g., at least two inpatient episodes within the last 12 months. This study revealed that the Health Diary telemonitoring system combined with a specialized HBHC unit significantly decreased the need for hospital care in elderly patients with advanced HF or COPD without increasing total healthcare costs [13].

Remote monitoring projects are also being developed to improve the management of geriatric syndromes, dealing mainly with the risk of falls [16,17] and the monitoring of neuro and psychobehavioral disorders [18,19].

Our solution is innovative as it allows for the remote monitoring of geriatric risks by way of non-intrusive medical sensors that collect and send the patient’s physiological data as well as questionnaires integrated into the MyPredi™ platform. Most of the work described in the scientific literature focuses on a single geriatric risk, e.g., the risk of falls. Our remote monitoring solution provides for a comprehensive evaluation of elderly patients. Thanks to the MyPredi™ remote monitoring platform, patients benefit from personalized medical follow-up that allows caregivers to prevent acute deteriorations in their condition. GER-e-TEC is a unique and innovative project. In fact, to the best of our knowledge, it is the only remote monitoring platform designed to help prevent the deterioration of geriatric syndromes. The platform makes it possible to detect the early warning signs of a deteriorating condition and sends alerts to the patient’s medical team. The daily medical monitoring of patients, based on personalized protocols set up by the medical staff, is performed by healthcare teams (nurses and caregivers) on the MyPredi™ platform. This monitoring includes measurements taken by sensors and questionnaires completed online. If a patient’s condition is at risk of deteriorating, an alert is sent to a medical coordination unit (nurses and doctors) so appropriate actions can be taken.

With MyPredi™, patients benefit from personalized and preventive care that improves their quality of life. This includes multidimensional care and the monitoring of several indicators that are not addressed by other platforms, such as the risk of constipation, dehydration, iatrogenesis, pain, infections associated with Covid-19, and sleep disorders. Our project arose from the need to improve care provided in nursing homes by combining digital transformation, the needs of the elderly, and the five P’s of medicine (predictive, preventive, personalized, participatory, and purpose-driven).

Our work has limitations, and we acknowledge that the remote monitoring system put in place can be improved. Indeed, the main limitation of our work lies in the number of alerts issued by our remote monitoring system for the 36 patients included. It would be advisable to conduct some smoothing in order not to saturate the system and to avoid interventions that are useless or redundant, as this has the consequence of increasing the anxiety levels of the elderly patients and their families. Smoothing is currently underway, in coordination with the scientific committee steering the study (geriatricians, cardiologists, internists, and the Predimed team managing the platform). In addition, neuropsychobehavioral disorders and mental confusion have not been specifically studied. They remain a real problem for the elderly and, in particular, the institutionalized elderly. Thus, our remote monitoring system will be enriched with a CAM (Confusion Assessment Method) scale [20] for the risk of “confusion” as well as an NPI (Neuropsychiatric Inventory) scale [21], which will be used during the second phase of our study that will begin in early December 2020. The “iatrogenism” risk will also be consolidated by the use of the STOPP/START version 2 scale [22].

## 12. Conclusions

This proof of concept will allow us to approve our technological processes and evaluate the ergonomics of the system and its use by the hospital care team. The next phase of the project will begin in December 2020 and will consist of a prospective, longitudinal, monocentric study to demonstrate, in terms of evidence-based medicine, how a personalized and standardized remote monitoring solution can improve the quality of life of elderly polypathological and polymedicated patients.

## Figures and Tables

**Table 1:** Remote monitoring of geriatric risks in the GER-e-TEC study.

Geriatric Risk	Connected Sensors/Questionnaires	Frequency
Hemodynamic data (hypertension/hypotension–tachycardia/bradycardia–oxygen desaturation/infections)	Sphygmomanometer–pulse oximeter–thermometer	Three times per day
Heart failure	Questionnaire	Daily
Constipation	Questionnaire	Twice a day
Risk of bed rest	Questionnaire Pedometer	Daily Daily
Pain	Questionnaire	Daily
Dehydration	Questionnaire Biological sensors (natremia–kaliemia–creatinine)	Daily Twice a week
Sleep quality	Pedometer	Day and night
Physical activity	Pedometer Questionnaire	Daily Daily
Diabetes	Glucometer	Three times per day
Iatrogenism	Questionnaire	On admission and once during hospitalization
Malnutrition	Balance Biological sensor (albumin)	Twice a week Once during hospitalization

**Table 2:** Detailed questionnaires included in MyPredi™ platform.

Geriatric Risk	Description of the Questionnaire
Heart Failure	- Does the patient have edema? - Does the patient have dyspnea? - Is the patient coughing? - Does the patient have orthopnea?
Constipation	- Number of stools in the morning - Number of stools in the evening
Risk of bed rest/Physical activity	- Is the patient on bed rest for more than 50% of the day?
Dehydratation	- Daily water volume?
Pain	- Quantification of pain according to the visual analogue scale rated from 0 to 10 - Quantification of pain according to the verbal rating scales, “What is the level of your pain at the moment?” 0 = None 1 = Mild 2 = Moderate 3 = Strong 4 = Worst pain - Quantification of pain according to the Algoplus scale in all situations in which reliable self-assessment is not feasible (e.g., those with difficulty communicating verbally): - 1. Facial expressions - Frowning, grimacing, wincing, clenched teeth, - unexpressive - 2. Look - Inattentive, blank stare, distant or imploring, - teary-eyed, closed eyes - 3. Complaints - “Ow-ouch”, “that hurts”, groaning, screaming - 4. Body position - Withdrawn, guarded, refuses to move, frozen - posture - 5. Atypical behaviors - Agitation, aggression, grabbing onto something - or someone.
Iatrogenism	- Iatrogenism and dehydration - Iatrogenism and cardiovascular system - Iatrogenism and undernutrition - Iatrogenism and confusion - Iatrogenism and fall - Iatrogenism and anticoagulants - Iatrogenism and diabetes

**Table 3:** Details of iatrogenic questionnaires.

Details of Iatrogenic Questionnaires	
Dehydration	• Diuretics • Laxatives • Anti-cyclicals • Selective serotonergic reuptake inhibitors • Proton pump inhibitors • ACE inhibitors • Carbamazepine • Valproate
Cardiovascular system	• Diuretics • ACE inhibitors, Sartan • Digoxin • Beta-blockers • Calcium channel blockers • Nitro derivatives • Antiarrhythmics
Confusion	Sedatives/hypnotics Benzodiazepines Antiepileptics Antiparkinsonians Anticholinergics Corticosteroids Central antihypertensive drugs Anti-arrhythmics Nonsteroidal anti-inflammatory drugs Antibiotics Analgesics Oral hypoglycemic agents
Fall	If taking nitrates, diuretics, converting enzyme inhibitors, antipsychotics, central antihypertensive drugs, tricyclic antidepressants, dopathotherapy (risk of fall by orthostatic hypotension) If taking antiarrhythmics, digitalis, hypokalaemic diuretics, spironolactone (risk of fall due to arrhythmia/conduction disorder) If taking insulin and sulfonylureas (risk of fall due to hypoglycemia) If taking anticoagulants, antiaggregants (risk of falling through anemia) If taking benzodiazepines, antiepileptics, antipsychotics (risk of falling due to disturbance of vigilance) If taking antiparkinson drugs, tricyclic antidepressants, lithium, anticholinergic drugs, carbamazepines, aminoglycosides (risk of fall due to delirium) If taking dopathotherapy, antipsychotics (risk of falling due to abnormal movements) If taking antipsychotics, metoclopramide, antihistamines (risk of fall due to parkinsonian syndrome).
Diabetes	Treatment with oral antidiabetics Treatment with insulin therapy
Malnutrition	Metronidazole, sulfonamides (risk of malnutrition by dysgeusia) Benzodiazepines, diuretics, antipsychotics (risk of undernutrition by dry mouth) Dopathotherapy, morphine, antibiotics, digoxin (risk of malnutrition through nausea) Morphine, calcium channel blockers, iron treatment (risk of undernutrition by inducing constipation) Digoxin, dopathotherapy, selective serotonin reuptake inhibitors (risk of undernutrition by anorectic behavior)
Anticoagulants	Taking anticoagulants If YES, existence of an association: pyrazole non-steroidal anti-inflammatory drugs Aspirin in high dose, antiplatelet agents Antibiotics (fluoroquinolones, macrolides, cyclins, cotrimoxazole, certain cephalosporins) Oral antifungals

**Table 4:** Technical characteristics of the MyPredi^TM^ platform sensors [3].

Dispositifs/Sensors	Characteristics
Balance	A&D Medical, Model: UC-352BLE Bluetooth: 4.0
Sphygomanometer	A&D Medical Model: UA-651BLE Bluetooth 4.0
Pulse oximeter	Jumper Model: JPD-500F Bluetooth: 4.0
Pedometer	Ecare Fit No model Bluetooth 4.0
Glucometer	FORA Advanced pro GD40 Model: TD-4272H/GD40h Bluetooth 4.0
Thermometer	Jumper Model JPD-FR302 Bluetooth 4.0

**Table 5:** Characteristics of the study population (*n* = 36).

Medical Characteristics (*n*,%)	
Medical History	
Heart deficiency	11 (30.5%)
Arterial hypertension	23 (63.8%)
Atrial fibrillation	7 (19.4%)
Coronary syndrome	7 (19.4%)
Pacemaker	6 (16.6%)
Obliterating arteriopathy of the lower limbs	6 (16.6%)
Sleep apnea syndrome	6 (16.6%)
Phlebitis/pulmonary embolism	2 (5.5%)
Dyslipidemia	10 (27.8%)
Obesity	4 (11.1%)
Diabetes	14 (38.9%)
Stroke	6 (16.6%)
Chronic renal deficiency	9 (25%)
COPD *	11 (30.5%)
Solids neoplasms	4 (11.1%)
Hematological malignancy	3 (8.3%)
Cirrhosis	2 (5.5%)
Peptic ulcer	7 (19.4%)
Hepatitis	4 (11.1%)
Hypothyroidism	3 (8.3%)
Connectivities	4 (11.1%)
Cognitive disorder	9 (25%)
Causes of hospitalization	
Pulmonary infection	17 (47.2%)
Acute heart failure	10 (27.8%)
Phlebitis/pulmonary embolism	1 (2.8%)
Urinary infection	2 (5.5%)
Anemia	3 (8.3%)
COPD * exacerbation	2 (5.5%)
Stroke	1 (2.8%)
Acute renal deficiency	2 (5.5%)
Acute dermohypoderma	2 (5.5%)
Confusion	2 (5.5%)
Keeping at home difficult	1 (2.8%)
Sigmoiditis	2 (5.5%)
Melaena	1 (2.8%)
Macroscopic hematuria	1 (2.8%)
Treatment	
Antihypertensives	33 (91.6%)
Beta blockers	20 (55.5%)
ACE inhibitors, Sartan	19 (52.7%)
Diuretics	21 (58.3%)
Calcium channel blockers	11 (30.5%)
anticoagulants	9 (25%)
Antiplatelet agents	16 (44.4%)
Statins	15 (41.7%)
Oral antidiabetics	7 (19.4%)
Insulin therapy	8 (22.2%)
Benzodiazepines	9 (25%)
Antipsychotics	6 (16.7%)
Antidepressant	2 (5.5%)
Proton pump inhibitors	13 (36.1%)
Antiarrhythmics	2 (5.5%)

* COPD: Chronic obstructive pulmonary disease.

**Table 6:** Collected data from sensors and questionnaires (*n* = 36 patients).

Data from Sensors and Questionnaires (Mean ± Standard Derivation)	
Arterial pressure	105.70 mm Hg (± 8.1 mm Hg)
Heart rate	77.6 bpm (± 15 bpm)
Oxygen saturation	96.5% (± 21)
Blood glucose level	124.3 mg/L (± 86 mg/L)
Weight	75.1 kg (± 23.1 kg)
Temperature	36.7 °C (± 0.6 °C)
Physical activity	925.4 steps per day (± 1280.8 steps per day)
Daily activity index	13.9% (± 14.1%)
Amount of sleep	500.3 min per day (± 206 min)
Amount of light sleep	139.8 min per day (± 144.4 min)
Amount of deep sleep	358.8 min per day (± 159.2 min)
Water volume	947.3 mL (± 386.4 mL)
Stool frequency	0.6 stools per day (± 0.6)
VAS pain score	1.2 (± 0.3)
VRS pain score	0.6 (± 0.2)
Algoplus	7.5 (± 2.3)
Albumin level	35.2 g/L (4.1 g/L)
Natremia	136.2 mmol/L (± 3.6 mmol/L)
Kalemia	4.2 mEq/L (± 0.6 mEq/L)
Creatinine level	87.3 µmol/L (± 30.2 µmol/L)
INR *	2.5 (± 1.4)

* Based on only two International Normalized Ratio (INR) measurements.

**Table 7:** Total alerts emitted per risk group and geriatric risk (“low”, “medium”, and “critical”).

Geriatric Syndromes	Alerts Total	Low Alerts	Moderate Alerts	Critical Alerts
Bed rest	192	192 (100%)	0	0
Tachy–bradycardia	28	0	0	28 (100%)
Malnutrition	60	0	51 (85%)	9 (15%)
Pain	137	0	137 (100%)	0
Hyperthermia	8	0	8 (100%)	0
Hypo- and hyperkalemia	66	0	66 (100%)	0
Hypo- and hypernatremia	9	0	9 (100%)	0
Hypo- and hypertension	274	0	0	274 (100%)
Iatrogenesis	211	91 (43.1%)	31 (14.7%)	89 (42.2%)
Heart failure	336	96 (28.6%)	29 (8.6%)	211 (62.8%)
Dehydration	290	222 (76.6%)	68 (23.4%)	0

**Table 8:** Alerts and recommendations issued for the geriatric risks studied.

	Alert Emitted *n*	Recommendations *n*
Risk related to iatrogenesis (+)	211	0
No risk related to iatrogenesis (−)	425	289
Risk of bed rest (+)	192	0
No risk of bed rest (−)	0	0
Risk of constipation (+)	0	0
No risk of constipation (−)	67	67
Risk of decompensated heart failure (+)	336	0
No risk of decompensated heart failure (−)	0	0
Risk of pain (+)	137	0
No risk of pain (−)	0	0
Risk of dehydration (+)	290	0
No risk of dehydration (−)	45	45
Risk of alteration in heart rate (+)	28	0
No risk of alteration in heart rate (−)	1	1
Risk of malnutrition (+)	60	0
No risk of malnutrition (−)	69	55

**Table 9:** Sensitivity, specificity, and positive and negative predictive values for alerts from the MyPredi™ remote monitoring platform.

	Decompensated Heart Failure	Pain	Dehydration	Brady- and Tachycardia	Constipation	Bed Rest	Malnutrition	Iatrogenesis
Sensitivity	100%	100%	100%	100%	-	100%	100%	100%
Specificity	-	-	50%	50%	50%	-	44%	40%
Positive predictive value	100%	100%	87%	97%	-	100%	47%	33%
Negative predictive value	-	-	100%	100%	100%	-	100%	100%

## References

[1] (2019). Institut National de la Statistique et des Etudes économiques (France), Plazaola J de, Rignols, E. Tableaux de L’économie Française. https://www.insee.fr/fr/statistiques/3676717?sommaire=3696937.

[2] Morley J.E., Rolland Y., Tolson D., Vellas B. (2011). The time has come to enhance nursing home care. Arch Gerontol. Geriatr..

[3] Zulfiqar A.-A., Noël L.V., Zulfiqar O.-A., Hajjam M., Courbon Q., Esteoulle L., Geny B., Talha S., Letourneau D., Hajjam J. (2020). e-Health: A Future Solution for Optimized Management of Elderly Patients. GER-e-TEC™ Project. Medicines.

[4] Andrès E., Talha S., Benyahia A., Keller O., Hajjam M., Moukadem A., Dieterlen A., Hajjam J., Ervé S., Hajjam A. (2016). Experimentation of an e-platform to detect situations at risk of cardiac impairment (platform E-care) in an internal medicine unit. Rev. Med. Interne.

[5] Gokalp H., De Folter J., Verma V., Fursse J., Jones R., Clarke M. (2018). Integrated Telehealth and Telecare for Monitoring Frail Elderly with Chronic Disease. Telemed. e-Health.

[6] Jourdain P., Desnos M., Juillière Y. (2014). Mise en place d’une Plateforme Interactive Médecin Patients Santé (PIMPS) basée sur une auto mesure à domicile d’un biomarqueur dans l’insuffisance cardiaque chronique ambulatoire. Eur. Res. Telemed. Rech. Eur. Télémédecine.

[7] Biannic C., Coutance G., Calus J., Belin A., Loiselet P., Michel L., Pradère G., Delmas P., Grollier G., Sabatier R. (2012). Suivi éducatif à domicile dans l’insuffisance cardiaque par télémédecine. Étude multicentrique bas normande randomisée. Résultats préliminaires. Eur. Res. Telemed. Rech. Eur. Télémédecine.

[8] Rubel P., Fayn J., Nollo G., Assanelli D., Li B., Restier L., Adami S., Arod S., Atoui H., Ohlsson M. (2005). Toward personal eHealth in cardiology. Results from the EPI-MEDICS telemedicine project. J. Electrocardiol..

[9] Mabo P., Victor F., Bazin P., Ahres S., Babuty D., Da Costa A., Binet D., Daubert J.-C. (2012). A randomized trial of long-term remote monitoring of pacemaker recipients (The COMPAS trial). Eur. Hear. J..

[10] Guédon-Moreau L., Lacroix D., Sadoul N., Clémenty J., Kouakam C., Hermida J.-S., Aliot E., Boursier M., Bizeau O., Kacet S. (2013). A randomized study of remote follow-up of implantable cardioverter defibrillators: Safety and efficacy report of the ECOST trial. Eur. Hear. J..

[11] Franek J. (2012). Home Telehealth for Patients with Chronic Obstructive Pulmonary Disease (COPD). Ont. Health Technol. Assess. Ser..

[12] Antoniades N.C., Rochford P.D., Pretto J.J., Pierce R.J., Gogler J., Steinkrug J., Sharpe K., McDonald C. (2012). Pilot Study of Remote Telemonitoring in COPD. Telemed. e-Health.

[13] Lyth J., Lind L., Persson H.L., Wiréhn A.-B. (2019). Can a telemonitoring system lead to decreased hospitalization in elderly patients?. J. Telemed. Telecare.

[14] Andrés E., Meyer L., Zulfiqar A.-A., Hajjam M., Talha S., Bahougne T., Ervé S., Hajjam J., Doucet J., Jeandidier N. (2019). Mise au point sur les projets de recherche dans le domaine de la télémédecine dans le diabète, avec un focus sur les projets de télésurveillance 2.0. Méd. Mal. Métaboliques.

[15] Guan K., Shao M., Wu S. (2017). A Remote Health Monitoring System for the Elderly Based on Smart Home Gateway. J. Healthc. Eng..

[16] Piau A., Mattek N., Crissey R., Beattie Z., Dodge H., Kaye J. (2020). When Will My Patient Fall? Sensor-Based In-Home Walking Speed Identifies Future Falls in Older Adults. J. Gerontol. Ser. A Boil. Sci. Med. Sci..

[17] Vermeulen J., Neyens J.C., Spreeuwenberg M., Van Rossum E., Boessen A.B.C.G., Sipers W., De Witte L., Pilling M., Pfortmueller C. (2015). The Relationship Between Balance Measured with a Modified Bathroom Scale and Falls and Disability in Older Adults: A 6-Month Follow-Up Study. J. Med. Internet Res..

[18] Franco G.C., Gallay F., Berenguer M., Mourrain C., Couturier P. (2008). Non-invasive monitoring of the activities of daily living of elderly people at home—A pilot study of the usage of domestic appliances. J. Telemed. Telecare.

[19] Piau A., Lepage B., Bernon C., Gleizes M.P., Nourhashemi F. (2019). Real-Time Detection of Behavioral Anomalies of Older People Using Artificial Intelligence (The 3-PEGASE Study): Protocol for a Real-Life Prospective Trial. JMIR Res. Protoc..

[20] Antoine V., Belmin J., Blain H., Bonin-Guillaume S., Goldsmith L., Guerin O., Kergoat M.-J., Landais P., Mahmoudi R., Morais J.A. (2018). The Confusion Assessment Method: Transcultural adaptation of a French version. Rev. Epidemiol. Sante Publique.

[21] Cummings J.L., Mega M., Gray K., Rosenberg-Thompson S., Carusi D.A., Gornbein J. (1994). The Neuropsychiatric Inventory: Comprehensive assessment of psychopathology in dementia. Neurology.

[22] Palchik V., Bianchi M., Colautti M., Salamano M., Pires N., Catena J.M., Dolza M.L., Tassone V., Lillini G., Paciaroni J. (2020). Pharmaceutical care for older adults. Application of STOPP-START criteria. J. Healthc. Qual. Res..

